# *“I'm luckier than everybody else!”*: Optimistic bias, COVID-19 conspiracy beliefs, vaccination status, and the link with the time spent online, anticipated regret, and the perceived threat

**DOI:** 10.3389/fpubh.2022.1019298

**Published:** 2022-11-15

**Authors:** Alexandra Maftei, Cosmina Elena Petroi

**Affiliations:** Faculty of Psychology and Education Sciences, Alexandru Ioan Cuza University, Iaşi, Romania

**Keywords:** COVID-19, conspiracy beliefs, vaccination, threat, anticipated regret

## Abstract

The catastrophic wave in the fall of 2021 drove Romania to the top of the list of dangerous COVID-19 infections, with the highest mortality rate in Europe. At the same time, Romania had one of the lowest vaccination rates. In this context, the present research aimed to explore the link between vaccination intention/status, optimistic bias, COVID-19 conspiracy beliefs, the time spent online, and vaccination (anticipated) regret. Our convenience sample was formed by 408 adults aged 18–63 years (*M* = 22.11, *SD* = 6.18, 69.9 % females), who were distributed into four groups: (1) non-vaccinated who definitely refused COVID-19 vaccination, (2) non-vaccinated who considered COVID-19 vaccination, (3) non-vaccinated who reported their absolute willingness to COVID-19 vaccination, and (4) people who were COVID-19 vaccinated. We conducted our analyses separately, depending on these groups (i.e., vaccination intentions/status). Data were collected using an online questionnaire between November 10, 2021, and January 03, 2022. In our cross-sectional approach, following correlation and ANOVA analyses, among the observed patterns were (1) the significant negative relation between optimism bias and the perceived COVID-19 threat; (2) the positive link between anticipated regret, post-vaccination regret, age, and conspiracy beliefs. We discuss our findings considering their contribution to health policies and practices.

## Introduction

Toward the end of 2019, the WHO (1) Headquarters was informed about cases of pneumonia of unknown etiology identified in the city of Wuhan, Hubei Province in China. Furthermore, the World Health Organization declared an international pandemic on March 11, 2020, after the new SARS-CoV-2 virus quickly spread to almost all countries around the globe (2). COVID-19 is a respiratory infection whose severity ranges from asymptomatic to severe and fatal disease. At the time of writing (August 2022), there have been more than 3 million confirmed cases of COVID-19 and over 66.000 deaths in Romania. Concerning the COVID-19 vaccination, more than 16 million vaccine doses have been administered (1).

However, at the time this research was conducted—at the end of 2021—Romania was ranked penultimate place in Europe, before Bulgaria, regarding the vaccination rate against COVID-19 (3). This was intriguing since, during the same time, Romania reported the highest mortality rate in Europe, with more than 500 deaths per day (4). Thus, it is essential for public health communication, strategies, and practices in the global fight against COVID-19 that we understand the motivational roots of vaccine hesitancy, one of the most controversial issues discussed in the past year.

Beliefs in COVID-19 conspiracy theories (CTs), lower educational levels, inadequate knowledge of COVID-19, younger age, and female gender are among the most common factors that indicate a refusal to vaccinate [see (5, 6) for reviews]. Factors that predict COVID-19 vaccination acceptance include the (high) perceived risk of COVID-19, an older age, trust in scientific experts, and accurate general information related to the COVID-19 vaccine (7, 8). In addition to these documented factors, the present research also aimed to explore the role of some less explored factors, i.e., optimistic bias, the time spent online, and anticipated regret when discussing COVID-19 vaccination intentions and status.

## Risk perception and response

Given the magnitude of the health crisis caused by COVID-19, the factors underlying preventive behaviors and compliance with protective measures become highly important. According to the Health Belief Model [HBM (9)]. and the Theory of Planned Behavior [TPB (10)], individuals adopt behaviors to minimize the threat of a disease when they perceive themselves to be more susceptible to developing that disease, and counteracting the disease would have severe consequences (11). In the case of the COVID-19 pandemic, individuals are more likely to engage in preventive behaviors if the severity and perceived susceptibility are high (11–15).

From the Health Beliefs Model perspective, the perceived threat is a significant driver of people's preventive actions. Specifically, the perception of a threat is positively related to individuals' intention to take protective actions (16). Furthermore, the perceived threat was recently linked to TBP components, and results showed that the perceived threat could predict behavioral intentions related to COVID-19 when mediated by attitudes and social norms (17). Regarding people's intentions (prospective behaviors), according to the Theory of Planned Behavior (10), behavior is predicted by intention, and intention is influenced by attitudes toward the behavior, subjective norms, and perceived personal control.

In the context of the COVID-19 pandemic, it has been observed that the higher the perceived threat, the more individuals seem to adhere to government measures and favor compliance with instructions aimed at avoiding contamination and the spread of the virus (13, 18, 19). Moreover, regarding vaccination, several studies suggested that high levels of perceived threat might have a direct effect on vaccination intention but also an indirect effect by influencing the decrease in beliefs related to conspiracy theories [e.g., (20, 21)].

## Optimistic bias

An individual's evaluation of risk vulnerability, risk severity, treatability of a malady, and the viability of preventive actions represents a series of components from the field of health psychology related to health-promoting behavior that belongs to risk perception (22). Sometimes human beings have a remarkable tendency to see the “fuller side of the glass” in everyday life, distorting their own risk in situations that could put them in danger. This phenomenon is known as *optimism bias* or *unrealistic optimism*, and according to Weinstein (23), it usually appears when an individual perceives their own risk to be lower than others. More precisely, in the case of this bias, the person's perception that their own risk of experiencing negative situations is lower than others leads to experiencing more positive conditions than others (24).

In previous studies, optimism bias has been associated with several risk behaviors, such as smoking, excessive alcohol consumption (25), and coronary heart disease (26). Regarding the COVID-19 pandemic, optimism bias has been associated with a lower perceived risk of infection (11, 27) and poorer adherence to prevention behaviors like wearing masks and social distancing (24, 28, 29). Regarding vaccination, it has been suggested that optimism bias may negatively influence vaccination intentions because people subject to optimism bias do not believe they need the vaccine as long as their risk of infection is low (30). However, previous studies have not found, until the present, a significant association between optimism bias and COVID-19 infection rates (31, 32).

## Conspiracy beliefs and COVID-19 vaccination

The COVID-19 conspiracy beliefs and theories generally promote the idea that the virus is not contagious and results from laboratory manipulations created to profit by distributing new vaccines (33). People's beliefs in conspiracy theories related to COVID-19 are an essential factor often negatively associated with the engagement in preventive behaviors and positively with pseudoscientific practices (33–35). For example, in a study by Maftei and Holman (20), personal compliance with lockdown rules was higher among participants who did not have convictions of possible conspiracies.

Moreover, previous research indicated that conspiratorial ideas discourage vaccination and influence negative attitudes toward vaccination (36–42). According to Maftei and Holman (43), among people who usually strongly believe in conspiracy ideas (e.g., the virus does not exist; governments invented the pandemic; the flu or even a product of Big Pharma), beliefs in conspiracy theories partially mediated the relationship between perceived threat and willingness of participants to vaccinate.

## Does the time spent online matter?

During this COVID-19 global health crisis, large-scale misinformation has significantly impacted the population's reluctance to vaccinate through relatively unregulated and decentralized platforms (44). Frequent exposure to negative information about COVID-19 vaccines on social media was associated with a lower vaccination rate (42, 45). For example, Ghaddar et al. (39) observed that a third of the sample, which showed a low vaccination rate, were dependent on social networks such as WhatsApp, Facebook, or Instagram and used them as primary sources of information. A significant positive relationship was also observed between vaccination hesitancy and frequent use of social networks such as Snapchat and TikTok; however, the strongest association was with excessive use of YouTube (44).

At the same time, other studies suggested a significant positive relationship between frequent exposure to social media content, interpersonal discussions, and vaccination intentions (46, 47). In addition, the excessive use of content on social networks was positively associated with a positive change in prevention behaviors and with obtaining the emotional, social, and informational support people need in this delicate period (46, 48). Thus, the findings in this area are mixed and call for further research.

## Vaccination anticipated and subsequent regret

According to the Regret Theory (49, 50), people anticipate the feelings they might experience when the outcome of a decision becomes obvious (50). Thus, analyzing the possible negative consequences of a decision that must be taken could trigger the appearance of anticipated regret (32). Anticipated regret is composed of anticipated regret for action and anticipated regret for inaction (49). The difference between the two in the context of the COVID-19 vaccination is that anticipated regret for vaccination negatively predicts the intention to vaccinate compared to anticipated regret of not vaccinating, which is a positive predictor of it (51, 52).

Concerning the COVID-19 pandemic, anticipated regret seems to be a significant predictor of hesitancy toward the COVID-19 vaccine (32, 53). This relationship is also supported by previous research on vaccination; for example, in the case of HPV vaccination, it was observed that anticipated regret for not vaccinating was a significant predictor of vaccination intention, and its ratings are higher than those of regret for vaccination (54, 55).

Regret aversion guides individuals' actions even after the decision is made and the action taken because the regret caused by actual negative feedback on foregone outcomes can influence subsequent decision-making (56). Thus, the negative result of a decision that triggers the experience of post-decisional regret can change how a person behaves when faced with another similar decision (57). In several studies, subsequent regret has been associated with psychological stress, depression, and anxiety, concerning health risk decisions (58, 59).

In the context of the COVID-19 pandemic, Luo et al. (47) observed that respondents with higher post-decisional regret scores were less willing to receive the booster dose. The results of this study indicate that regret over previous decisions could significantly mediate the impact of post-vaccination adverse reactions on willingness to take the booster dose.

## The present study

Previous research suggests that several psychological elements identified by the Theory of Planned Behavior [TPB (10)] influence health-related behaviors [including COVID-19 vaccination (43)]. TPB states that attitude, subjective norms, and perceived behavioral control shape people's behavioral intentions. Adiyoso and Wilopo (17) suggested that threat perception might predict behavioral intentions related to COVID-19 when mediated by attitudes and social norms, in line with earlier findings on risk perception in health-related circumstances (60). At the same time, high levels of perceived threat might directly impact vaccination acceptance and intentions, but they also indirectly influence the decrease in beliefs related to conspiracy theories [e.g., (21, 43). Also, optimism bias may negatively influence vaccination intentions (30) due to a low COVID-19 threat perception (11). Next, previous studies suggested that anticipated vaccination regret negatively predicted vaccine reluctance (32, 53), and non-vaccination regret positively predicted COVID-19 vaccine acceptance (51, 52). Also, exposure to negative information about frequent social network use was associated with a lower vaccination rate (39, 44), though the findings in this area are mixed (46, 47).

Thus, the main assumptions of the present study were the following: *H1*. There would be significant negative associations between optimism bias and the perceived threat, regardless of participants' vaccination status and intentions; *H2*. There would be a significant positive association between optimism bias and anticipated regret for vaccination in the case of participants who have not yet been vaccinated; *H3*. There would be a significant negative association between optimism bias and anticipated regret for not vaccinating in the case of participants who have not yet been vaccinated and *H4*. There would be a significant positive association between optimism bias, conspiracy beliefs, the perceived threat, and the time spent online, regardless of vaccination status.

## Methods

### Participants and procedure

Four hundred and eight adults formed our final convenience sample from Romania aged 18–63 years (*M* = 22.11, *SD* = 6.18). Of the total respondents to the study, 69.9% were female, 29.4% were male, and 0.7% reported other genders. According to Lin et al. (61), the age groups our participants fall into are the *youth* group (18–47) and the *middle-aged* group (48–63) (please see Table 1a). Of the 408 participants, 2.2% (*N* = 9) had a secondary school diploma, 77% (*N* = 314) a high-school diploma, and 20.8% (*N* = 85) had a university degree. Twenty-three participants from the initial sample were excluded due to age criteria (i.e., all participants had to be over 18), whereas another was removed because they disagreed with data processing. There were no other inclusion/exclusion criteria.

**Table 1a:** Descriptive statistics for the main variables (overall sample, *N* = 408).

**Variable**	**M**	**SD**	**Min**	**Max**	**Skewness**	**Kurtosis**
Age (overall, *N* = 408)	22.13	6.16	18	63	–	–
Age group: 18–47 (*N* = 402)	21.61	4.75	18	46	–	–
Age group: 48–63 (*N* = 6)	52.50	5.75	48	63	–	–
Time spent online	2.41	1.17	0	4	−0.05	−1.06
Perceived threat	15.66	5.37	4	28	−0.10	−0.48
Conspiracy beliefs	25.71	12.21	6	60	0.38	−0.45
Anticipated regret (for vaccination)	2.25	1.51	1	5	0.80	−0.89
Anticipated regret (for non-vaccination)	4.53	2.29	1	7	−0.38	−1.36
Optimism bias	9.20	2.84	2	14	−0.32	−0.22

The present study's data were collected online through an online questionnaire and distributed *via* social media platforms and communication groups (Facebook, Instagram, Messenger, and WhatsApp). We targeted Romanian-only groups (i.e., the items were all written in Romanian). The research link was accompanied by information regarding the purpose of the research (i.e., the exploration of the factors related to the COVID-19 general response). The data collection period was between November 10, 2021, and January 03, 2022.

All participants voluntarily took part in this study, and they were informed that the information they provided would remain anonymous and confidential and that they could retire from this study at any time. The time needed to complete the questionnaire was around 15 min. The research was conducted following the Helsinki Declaration ethical criteria and the ethical research requirements approved by the institutional board of the authors' institution.

### Measures

#### COVID-19 vaccination intentions/status (outcome variable)

Participants' intentions to vaccinate were measured using an item targeting vaccination status, and the answer options were coded from 1 to 4, where 1 means *I have not been vaccinated against COVID-19 and I categorically exclude this possibility*, 2 means *I have not been vaccinated against COVID-19, but it is possible to do so*, 3 means *I haven't been vaccinated yet, but I'm sure I will*, and 4 means *I've already been vaccinated*. This measurement was previously used by Meyer et al. (62) to measure vaccination intentions/status.

#### COVID-19 conspiracy beliefs (exposure variable)

The COVID-19 conspiracy beliefs scale (63) consists of 6 items measured on a 10-point Likert scale ranging from 1 (*do not agree at all)* to 10 (*fully agree*). Example items include “*I believe the pharmaceutical industry is involved in the spread of the coronavirus*.” and “I *believe the coronavirus was intentionally made in a laboratory*.” High scores represent a high level of COVID-19-related conspiracy beliefs. The internal consistency indicated by Cronbach's alpha was 0.77.

#### COVID-19 threat perception (exposure variable)

The COVID-19 Threat Perception Scale (21) was used to measure threat perception. The instrument contains four items (i.e., “*To what extent are you currently worried about the spread of coronavirus*?”, and “*To what extent do you currently feel threatened by the spread of coronavirus*?”) measured on a 7-point Likert scale, ranging from 1 (*not at all*) to 7 (*very much*). High scores indicated a high level of perceived threat reported by the participants. The internal consistency indicated by Cronbach's alpha was good at 0.80. The instrument was previously used in a Romanian adult sample by Maftei and Holman (43), who reported a similar internal consistency (Cronbach's α = 0.86).

#### COVID-19 optimism bias (exposure variable)

We used two items to measure optimism bias, following the same procedure previously used by Wolff (32). The items measured relative perceived susceptibility and relative perceived probability of a serious prognosis: “*Compared to other Romanians of your age, what is the probability that you will be infected with COVID-19*?” and “*Compared to others Romanians of your age, what is the probability that you will experience severe symptoms following infection with COVID-19*?.” We used a 7-point Likert scale ranging from 1 (*extremely low*) to 7 (*extremely high*). To obtain the total score for this variable, we first reversed the items and then calculated the sum of the scores, with high scores representing a high level of optimism bias. Internal consistency indicated by Cronbach's alpha was 0.74.

#### COVID-19 vaccination anticipated regret (exposure variable)

We measured the anticipated regret for vaccination (i.e., *If I vaccinate against COVID-19, I might regret it*) and anticipated regret for not vaccinating (i.e., *If I don't vaccinate against COVID-19, I might regret it*), using the two items previously used by Wolff (32). We used a 7-point Likert scale ranging from 1 (*very unlikely*) to 7 (*very likely*). High scores represented high levels of anticipated regret for vaccination and anticipated regret for not vaccinating. Subsequent regret was introduced to measure the regret of people who had already been vaccinated against COVID-19. A single item measured this (e.g., “I got vaccinated against COVID-19, and I regret it”) using a 7-point Likert scale ranging from 1 (*absolutely not*) to 7 (*extremely much*). High scores indicated a high level of regret of subsequent vaccination.

#### Time spent online (exposure variable)

Participants' time spent online was measured using an item targeting the number of hours spent daily online, and the answer options were coded from 1 to 5, where 1 means 0–1 h, 2 means 1–3 h, 3 means 3–5 h, 4 means 5–7 h, and 4 means over 7 h. Thus, the higher the score, the higher the time spent online.

Finally, a demographic scale was used to assess participants' gender (self-reported), age, and education level. Using the forward-backward translation strategy, the scales were translated from English to Romanian (64). The minimal differences between the original and back-translated versions were reconciled, resulting in the final versions of each instrument.

### Statistical analysis

We used the SPSS 26.0 program to analyze our data. First, we computed the Skewness and Kurtosis values for our variables to assess the normality of the distributions (65), and we further used parametric tests (see Table 1a for the descriptive statistics of the variables).

We also computed the means and standard deviations for the main variables considering the participants' vaccination intention/status (see Table 1b).

**Table 1b:** Descriptive statistics for the main variables depending on participants' vaccination intention/status.

**Vaccination intention/status**				
**Variable**	**Definitely not (*N* = 54), M (SD)**	**I will consider it (*N* = 69), M (SD)**	**I will definitely get a vaccine (*N* = 15), M (SD)**	**I already got a vaccine (*N* = 270), M (SD)**
Optimism bias	9.38 (3.47)	8.84 (3.00)	8.60 (3.58)	9.30 (2.61)
Perceived threat	14.14 (6.32)	15.11 (5.42)	17.26 (5.72)	16.02 (5.08)
Conspiracy beliefs	36.96 (11.27)	30.07 (10.91)	29.14 (14.84)	22.15 (10.74)

Next, we examined the associations between the main variables (see Tables 2a–d), considering the vaccination status of the participants. Additionally, we also explored the potential gender differences concerning the primary variables in our study. Finally, we conducted One Way and Univariate ANOVA analyses to explore the potential interaction effects between gender and vaccination status concerning optimism bias, the perceived COVID-19 threat, and COVID-19 conspiracy beliefs.

**Table 2a:** Associations between the main variables (*N* = 54, participants who exclude vaccination).

**Variable**	**1**	**2**	**3**	**4**	**5**	**6**
1. Age	–					
2. Time spent online	−0.17	–				
3. Perceived threat	−0.04	0.04	–			
4. Conspiracy beliefs	0.22	0.09	0.17	–		
5. Anticipated regret (for vaccination)	0.08	0.15	0.11	−0.00	–	
6. Anticipated regret (for non-vaccination)	0.05	−0.05	0.07	0.10	−0.12	–
7. Optimism bias	0.02	0.10	−0.48^**^	−0.14	−0.08	−0.25

** p < 0.01, two-tailed.

**Table 2b:** Associations between the main variables (*N* = 59, participants who consider the possibility of vaccinating against COVID-19).

**Variable**	**1**	**2**	**3**	**4**	**5**	**6**
1. Age	–					
2. Time spent online	−0.43^**^	–				
3. Perceived threat	−0.05	0.26^*^	–			
4. Conspiracy beliefs	0.09	−0.11	0.00	–		
5. Anticipated regret (for vaccination)	−0.12	−0.06	0.11	0.44^**^	–	
6. Anticipated regret (for non-vaccination)	0.09	0.08	0.13	−0.08	−0.12	–
7. Optimism bias	0.00	−0.13	−0.44^**^	−0.19	−0.23	−0.35^*^

* p < 0.05, two-tailed.

** p < 0.01, two-tailed.

**Table 2c:** Associations between the main variables (*N* = 15, participants who will definitely vaccinate against COVID-19).

**Variable**	**1**	**2**	**3**	**4**	**5**	**6**
1. Age	–					
2. Time spent online	0.14	–				
3. Perceived threat	0.55^*^	0.13	–			
4. Conspiracy beliefs	0.56^*^	0.13	0.13	–		
5. Anticipated regret (for vaccination)	0.57^*^	−0.17	0.38	0.61^*^	–	
6. Anticipated regret (for non-vaccination)	−0.29	−0.33	−0.20	−0.05	−0.24	–
7. Optimism bias	−0.51	0.17	−0.62^*^	−0.37	−0.63^*^	0.35

* p < 0.05, two-tailed.

**Table 2d:** Associations between the main variables (*N* = 270, participants who were already vaccinated against COVID-19).

**Variable**	**1**	**2**	**3**	**4**	**5**
1. Age	–				
2. Time spent online	−0.28^**^	–			
3. Perceived threat	0.04	0.01	–		
4. Conspiracy beliefs	−0.06	−0.02	0.07	–	
5. Subsequent regret (for vaccination)	0.00	−0.04	−0.02	0.37^**^	–
6. Optimism bias	−0.12^*^	0.06	−0.32^**^	−0.10	−0.07

* p < 0.05, two-tailed.

** p < 0.01, two-tailed.

## Results

### Participants who excluded the possibility of vaccinating against COVID-19 (*N* = 54)

In the case of participants who definitely excluded the possibility of vaccinating against COVID-19, the only significant association we observed was between the perceived threat and optimist bias (*r* = −0.48, *p* < 0.001). More specifically, a higher perceived threat was associated with lower optimism bias.

### Participants who consider the possibility of vaccinating against COVID-19 (*N* = 59)

In the case of participants who were not vaccinated against COVID-19 but considered the possibility to do that, we found a negative association between the time spent online and age (*r* = −0.43, *p* < 0.001), and, as in the case of the first group (i.e., participants who excluded the possibility of vaccinating against COVID-19), higher optimism bias was associated with lower perceived COVID-19 threat (*r* = −0.44, *p* < 0.001). We also found positive associations between the time spent online and the perceived threat (*r* = 0.26, *p* = −0.03) and between anticipated vaccination regret and conspiracy beliefs (*r* = 0.44, *p* < 0.001). Thus, the higher the time spent online, the higher the perceived threat, and the higher the anticipated regret, the higher the conspiracy beliefs. Finally, optimism bias was negatively related to anticipated vaccination regret (*r* = −0.35, *p* = 0.003). Thus, the higher the optimism bias, the lower the anticipated regret.

### Participants who will definitely get vaccinated against COVID-19 (*N* = 15)

In the case of participants who were not vaccinated but reported their absolute intention to get vaccinated against COVID-19, we found positive associations between the perceived COVID-19 threat and age, *r* = 0.55, *p* = 0.03, i.e., the older the participants, the higher the perceived COVID-19 threat. Also, our data suggested negative links between the perceived threat and optimism bias (*r* = −0.62, *p* = 0.01), meaning that participants who perceived a higher COVID-19 threat also reported lower optimism bias. Next, we found that the higher the age, the higher the COVID-19 conspiracy beliefs (*r* = 0.56, *p* = 0.02) and the anticipated vaccination regret (*r* = 0.57, *p* = 0.02). In other words, older participants also reported higher conspiracy beliefs and anticipated vaccination regret. Furthermore, the results suggested that optimism bias was negatively associated with vaccination regret (*r* = −0.63, *p* = 0.01), i.e., the higher the optimism bias, the lower the regret.

### Participants who already got vaccinated against COVID-19 (*N* = 270)

In the case of participants who were vaccinated, our data suggested that age was negatively related to optimism bias (*r* = −0.12, *p* = 0.04) and the time spent online (*r* = −0.28, *p* < 0.001). In other words, in this group, older participants reported lower optimism bias and lower time spent online. Furthermore, a higher perceived COVID-19 threat was negatively associated with optimism bias (*r* = −0.32, *p* < 0.001), i.e., the higher the perceived threat, the lower the optimism bias. Finally, in this group, higher post-vaccination regret was associated with higher conspiracy beliefs (*r* = 0.37, *p* < 0.001).

### One-way ANOVA test results

We further conducted Anova One Way analyses to determine the potential differences based on vaccination intentions/status regarding optimism bias and the perceived COVID-19 threat. The analyses were performed using these variables since they comprised the common factor for the four analyses. The results suggested no significant differences between the groups when discussing optimism bias, *F*_(3,404)_ = 0.78, *p* = 0.50. However, we found marginally significant differences concerning the perceived COVID-19 threat, *F*_(3,404)_ = 2.55, *p* = 0.055. However, *post-hoc* Bonferroni analyses did not reveal any subsequent significant differences (all *p*-s > 0.05).

Next, we aimed to examine how conspiracy beliefs might predict participants' anticipated regret for vaccinating, anticipated regret for non-vaccination, or subsequent regret (following vaccination) in each group when moderated by age. We aimed to select the groups in which we previously observed significant links between these variables (that would further allow moderation analyses), i.e., the participants who consider the possibility of vaccinating against COVID-19 (*N* = 59) and participants who will definitely vaccinate against COVID-19, *N* = 15. However, given the number of participants in these groups, these analyses were not considered reliable (due to a very low statistical power level).

Finally, we explored the potential interaction effect between gender and vaccination status regarding optimism bias, the COVID-19 threat, and conspiracy beliefs. The results of the ANOVA Univariate analyses are summarized in Table 3. Our data suggested no interaction effects in any of the cases (all *p*-s > 0.05). Regarding optimism bias, the results suggested no significant main nor interaction effects, all p-s >0.05. Regarding the perceived COVID-19 threat, our data indicated that the only significant results were related to the main effect (and not the interaction effect) of gender, *F*_(8,407)_ = 4.34, *p*= 0.01, as well as vaccination status, *F*_(8,407)_ = 2.84, *p* = 0.03. However, the effect sizes were small in both cases, i.e., η^2^= 0.02 for both gender and vaccination status. Finally, regarding conspiracy beliefs, we found a large effect (η^2^ = 0.15) of vaccination status, *F*_(8,407)_ = 23.57, *p* < 0.001, but no interaction effect (*p* = 0.68).

**Table 3:** Univariate ANOVA test results for optimism bias, perceived threat, and conspiracy beliefs (vaccination status × gender).

**Optimism bias**		**Vaccination status × gender M (SD)**	**Test of between-subject effects**			
**Vaccination status**	**Gender**		
Group 1 (*N* = 54)	Female	9.27 (3.34)		*F*_(8,407)_	*p*	η^2^
	Male	9.81 (4.09)	Vaccination Status	0.52	0.66	0.004
Group 2 (*N* = 69)	Female	8.55 (3.02)	Gender	1.90	0.15	0.009
	Male	9.55 (2.91)	Vaccination status × gender	0.55	0.64	0.004
Group 3 (*N* = 15)	Female	7.75 (3.49)				
	Male	9.57 (3.69)				
Group 4 (*N* = 270)	Female	9.23 (2.62)				
	Male	9.48 (2.58)				
**Perceived threat**		**Vaccination status** × **gender M (SD)**	**Test of between-subject effects**			
**Vaccination status**	**Gender**		
Group 1 (*N* = 54)	Female	9.27 (3.34)		*F*_(8,407)_	*p*	η^2^
	Male	9.81 (4.09)	Vaccination Status	2.84*	0.03	0.02
Group 2 (*N* = 69)	Female	8.55 (3.02)	Gender	4.34*	0.01	0.02
	Male	9.55 (2.91)	Vaccination status × gender	0.83	0.47	0.006
Group 3 (*N* = 15)	Female	7.75 (3.49)				
	Male	9.57 (3.69)				
Group 4 (*N* = 270)	Female	9.23 (2.62)				
	Male	9.48 (2.58)				
**Conspiracy beliefs**		**Vaccination status** × **gender M (SD)**	**Test of between-subject effects**			
**Vaccination status**	**Gender**		
Group 1 (*N* = 54)	Female	9.27 (3.34)		*F*_(8,407)_	*p*	η^2^
	Male	9.81 (4.09)	Vaccination status	23.57**	0.00	0.15
Group 2 (*N*=69)	Female	8.55 (3.02)	Gender	0.40	0.67	0.002
	Male	9.55 (2.91)	Vaccination status × gender	0.49	0.68	0.004
Group 3 (*N* = 15)	Female	7.75 (3.49)				
	Male	9.57 (3.69)				
Group 4 (*N* = 270)	Female	9.23 (2.62)				

## Discussion

The catastrophic wave in the fall of 2021 drove Romania to the top of the list of dangerous COVID-19 infections, with the highest mortality rate in Europe. At the same time, Romania had one of the lowest vaccination rates. In this context, the present research aimed to explore the link between vaccination intention/status, optimistic bias, COVID-19 conspiracy beliefs, the time spent online, and vaccination (anticipated) regret.

### Optimism bias and COVID-19 vaccination

Our results suggested a significant negative association between optimism bias and the perceived threat in all four groups of participants. This result is consistent with those suggested by Garrett et al. (66), according to which optimism bias no longer appears when the perceived threat level is optimal, thus allowing a more accurate risk assessment or diminishing when an immediate threat is present in the environment. For example, Wise et al. (29) examined how a higher involvement in prevention behaviors is preceded by an increase in perceived personal risk and, respectively, a decrease in optimism bias.

Regarding the status and intentions to vaccinate against COVID-19, our results suggested that participants with a high level of the perceived threat and a low level of optimism bias seem to be more likely to get vaccinated, in line with previous studies in this area [e.g., (12, 15, 43)]. Furthermore, the present findings also align with a study carried out in Poland at the beginning of the pandemic [i.e., (67)], which evaluated participants' beliefs in three distinct moments regarding personal chances of personal contracting the virus. Dolinski and their collaborators observed a decrease in optimism bias and an intensification of the perceived personal risk among women the week after the announcement of the first COVID-19 infection due to the increase in the perceived threat level (67). Thus, our results seem to align with the general overview regarding the inversely proportional association between optimism bias and perceived threat regarding engagement in prevention behaviors such as COVID-19 vaccination.

### Optimism bias and the anticipated regret for vaccination

Furthermore, we found a significant negative association between the optimism bias and the anticipated regret for vaccination in the group of participants who reported they would definitely get vaccinated, which is a novelty brought by the present study, given its focus on four separate participant groups. Anticipated regret was suggested as a significant predictor of vaccination intention in several previous studies [e.g., (51, 68, 69)]. Also, according to the results of a study that aimed to examine the main factors of vaccine hesitancy from the perspective of HBM and TPB, anticipated regret was the most significant predictor of vaccination, with a high score of anticipated vaccination regret indicating a more negative attitude toward of vaccination (12).

However, when Chen and Yeh (70) examined the factors affecting the intention to engage in preventive behaviors, they did not find a significant moderating effect of optimism bias on anticipated action regret. Furthermore, according to the results presented by Wolf (32), anticipated regret for vaccination seems to be lower than anticipated regret for not vaccinating, and optimism may not predict vaccination intention. These explanations could support the non-significant associations obtained between optimism bias and anticipated regret in terms of participants who excluded vaccination and those who reported the possibility of getting vaccinated in the future. At the same time, Khayyam et al. (51) suggested that the perceived susceptibility to contracting COVID-19 mediated participants' regret concerning COVID-19 vaccination. Thus, these findings highlight the possibility of other variables influencing the associations between anticipated regret and vaccination and optimism bias and vaccination. Nevertheless, our results also indicated a negative association between optimism bias and anticipated regret, depending on vaccination intentions and status, which can be observed in various research and cultural contexts.

### Optimism bias and anticipated regret for not vaccinating

We also found a significant association between optimism bias and anticipated regret for not vaccinating in participants who considered getting vaccinated. The association between optimism bias and anticipated regret for not vaccinating has not been studied in the past in a similar context, and its analysis is a novelty of the present work. However, anticipated regret for not vaccinating was an important predictor of anti-COVID-19 vaccination intention in several studies (32, 71, 72). A significant example in this regard is represented by a longitudinal study on the Israeli population that analyzed several possible factors that might impact the intention to vaccinate against COVID-19 [i.e., (52)]. The results presented by the authors suggested that anticipated regret for not vaccinating might be a better predictor of vaccination intention than anticipated regret (52). Consistent with this idea, similar patterns were also observed in other contexts, such as HPV vaccination, where the anticipated regret for not vaccinating was also a significant predictor of vaccination intention (54, 55, 73).

Previous studies also suggested that people with a high level of anticipated regret for not vaccinating and a high level of optimism might be more likely to vaccinate (15, 52). At the same time, other studies suggested that anticipated regret was not a significant predictor of vaccination intention [e.g., (74)]. Furthermore, Wolff (32) suggested that anticipated regret for not vaccinating negatively predicted intention to vaccinate, this result being attributed to the idea that the lower the disadvantages of not vaccinating, the less socially accepted the side effects of vaccination will be. These findings might support and also explain the non-significant associations we found between optimism bias and anticipated regret for not vaccinating in the case of participants who excluded vaccination or were absolutely sure about their intention to vaccinate against COVID-19.

### Optimism bias and subsequent vaccination regret

Our findings also suggested no significant negative association between optimism bias and subsequent vaccination regret among participants who have already been vaccinated. This result might be explained by the fact that people interested in vaccinating against COVID-19 might have a higher desire to protect their family and community (75). Thus, even if individuals have a high optimism bias and perceive a low risk of infection for themselves, they may have been vaccinated to protect those around them, assessing the risk of others as higher. At the same time, this desire of people to protect their close ones could be amplified by the fact that participants with a high level of optimism bias evaluate their own risk of infection much higher for an acquaintance than for themselves, considering the possibility of being infected soon as more temporally distant for oneself than for other individuals (27). Furthermore, the perceived benefits might also be an additional significant predictor of intentions to vaccinate against COVID-19, as suggested by previous studies (52, 74). Therefore, a high level of perceived benefits may eliminate subsequent vaccination regret for people with a high level of optimism bias. More specifically, the non-significant association obtained between optimism bias and subsequent regret for vaccination could be due to a cause such as individuals' desire to protect their loved ones or the existence of a high level of perceived benefits. However, these potential explanatory mechanisms need to be explored in further studies.

At the same time, another pattern suggested by the present results was related to the positive link between anticipated regret, post-vaccination regret, and conspiracy beliefs. The pandemic is not over yet; according to WHO (1), new waves are coming with modified versions of the SARS-CoV-2 [The European Centre for Disease Prevention and Control (76)]. However, it seems that an adapted series of vaccines against the BA.4, BA.5, and BA.1 variants of the Omicron are waiting for approval, so we might likely have to re-vaccinate (76). In this context, exploring the underlying mechanisms regarding anticipated and subsequent regret concerning COVID-19 vaccination, especially those related to conspiracy beliefs, is essential.

### Optimism bias and time spent online

Our results suggested no significant positive association between optimism bias and time spent online across all four participant groups. This result is consistent with the results obtained in a study that analyzed the association between optimism bias and various factors in the context of the H1N1 flu; the authors observed an insignificant relationship between optimism bias and social media (77). In addition, previous studies suggested that being constantly exposed to the news was associated with a lower optimism bias in the context of perceived personal risk. In contrast, exposure to COVID-19-specific information was associated with a high optimism bias (78). At the same time, the negative news about COVID-19 vaccines presented on social media and low trust in the health system was associated with a lower level of vaccine acceptance (79). Furthermore, social media use seems also to be more strongly associated with conspiracy beliefs about the SARS-CoV-2 virus when conspiratorial thinking is heightened (80). Thus, the non-significant association observed in our study between the optimism bias and the time spent in the online environment could be explained by the fact that the media content watched would have a decisive role in this regard, and some people are predisposed to follow the information that aims to distort because of a high level of conspiratorial thinking. Thus, it is highly important to explore further the specific media contents that people are generally exposed to regarding health matters, to understand better the link between time spent online, which could also mean engagement in various activities such as academic or personal research, work-related activities, friends' online social gathering.

### Vaccination group differences

A novelty of the present study is also related to the fact that it also examined the differences between participants who excluded vaccination and participants who considered this option, those who were sure they would vaccinate, and those who have already been vaccinated in terms of the optimism bias, the perceived threat, and conspiratorial beliefs, differences that have not yet been explored in the context of the COVID-19 pandemic, to our knowledge (at least not in Romania). According to our results, there were no significant differences in optimism bias between any of the groups. These non-significant results are similar to some of the previous findings, according to which optimism bias was not a significant predictor of COVID-19 vaccination intentions (31, 32). A non-significant association between optimism bias and vaccination intention or adherence to preventive behaviors has also been observed for the H1N1 virus (77, 81). A possible explanation of these results could be related to the fact that a high perception of risk in close people could have influenced adherence to prevention behaviors such as vaccination. For example, exposure to friends' vaccination-related posts is positively associated with vaccination intention through positive affective responses (46). In addition, some people might resort to vaccination even if the level of perceived personal risk is low to protect the people around them (75).

Also, people seem to be more inclined to adhere to preventive behaviors if they understand that they are at risk, as measured by the susceptibility and perceived severity of the virus (11). However, a study that included participants with clinical conditions observed that while they showed a comparatively unrealistic optimism about their own infection compared to infecting others, this effect was not found for the risk of severe symptoms. Thus, it might be possible that some people show an optimism bias for perceived personal susceptibility but not for severity (82). Nevertheless, the optimism bias may have also been influenced by other aspects such as perceived risk from others, the desire to protect others, gender differences, or differences in perceived personal susceptibility or severity, which could be explored in future studies.

Furthermore, we found no significant differences in perceived threat between any groups. Comparative results were also obtained in a study that analyzed the hesitation of mothers regarding vaccination and the perceived threat influencing adherence to preventive behaviors such as wearing a mask but not the intention to vaccinate (83). Similarly, Bodas et al. (84) suggested that, while perceived threat did not predict vaccination intention, the importance of vaccination and perceived vaccine effectiveness were significant predictors (84).

An alternative explanation for our results could be related to the introduction of green certificates and restrictions for vaccinated people. The *green certificate* is a document that certifies that a person has either been vaccinated against COVID-19 or has had COVID-19 and recovered from the disease. In the European Union, during the coronavirus pandemic, a series of restrictions were introduced for people who did not hold such a certificate, such as travel restrictions, participation in various group events, or activities in closed spaces such as going to the cinema or gym. According to analyzes of vaccination rates following the introduction of these green certificates, there was an increase of 13 % in France, 6.2 % in Germany, and 9.7 % in Italy in terms of vaccination levels in the population after the introduction of green certificates (85). In Romania, starting October 10, 2021, following the adoption of Government Decision no. 1,090/2021, the daily vaccination rate increased from 52,815 vaccines administered on 9.10.2021 to 71,605 vaccines administered on 10.11.2021 according to the National Committee for the Coordination of Activities on Vaccination against COVID-19 (3). Thus, the non-significant differences obtained between participants who excluded vaccination and the other three groups of participants who were considering vaccination or have already done so in terms of perceived threat could be because perceived vaccine efficacy might be an important factor in the decision to vaccinate or could be caused by restrictions implemented by the state for unvaccinated people.

We also found significant differences between participants who excluded vaccination and participants who expressed the possibility of getting vaccinated in the future in terms of conspiracy beliefs, with the mean of people who excluded vaccination being significantly higher. These results are consistent with previous findings suggesting that conspiracy beliefs are a significant negative predictor of vaccination intention (39, 40, 42).

Contrary to our expectations, no significant differences were observed between participants who excluded vaccination and those who were sure they would vaccinate in terms of conspiracy beliefs. According to the previous research exploring the relationship between conspiratorial beliefs and vaccination intention, when the subjective norm is high (i.e., when the participants perceived that others close to them approved of vaccines), conspiratorial mentality no longer predicts vaccination intentions (86). Furthermore, estimated social norms were also positively associated with participants' intentions regarding the importance of getting a COVID-19 vaccine (87). Moreover, perceived disease risk and vaccine dangerousness have been observed to be mechanisms by which conspiracy theories can discourage vaccination (37). Thus, there may be other aspects that can influence vaccination statuses, such as vaccine efficacy or social norms, which can be discussed and examined in future studies.

### Gender differences

Finally, we found that the female participants from the group of participants who did not get a vaccine but considered the possibility scored higher than males on the perceived COVID-19 threat. Also, the female participants from the group of participants who were already vaccinated against COVID-19 scored higher than males on the perceived COVID-19 threat. These results align with previous findings that reported that women are generally more likely to report high levels of threat and fear of COVID-19 [e.g., (88)], as well as other similar infectious disease outbreaks [e.g., H1N1 (89); SARS-CoV-1 (90)]. Thus, our findings add to the literature that generally suggests that women are more likely to engage in preventive behaviors than men [e.g., (91)].

At the same time, female participants also scored higher than males regarding the COVID-19 conspiracy beliefs in the already vaccinated participants. This result contradicts previous findings from the early stages of the pandemic suggesting the opposite, i.e., males are more prone to COVID-19 conspiracy beliefs [e.g., (92)] or following the development of COVID-19 vaccines [e.g., (63)], as well as other studies suggesting that COVID-19 conspiracy operates similarly in men and women (93). However, our results also align with similar findings [e.g., (94)], highlighting the mixed data in this area and the need for future research to better clarify these potential gender differences. These results are important since they have implications for public health campaigns, given their useful input in shaping effective preventive health strategies, not only for COVID-19 but for future similar health crises.

## Implications of the present results

One of the novelties of this paper is that it explored the association between optimism bias and several key variables associated with vaccination intention, such as perceived threat and anticipated regret. In several previous empirical approaches, optimism bias was suggested as a possible predictor or explanatory mechanism of the intention to vaccinate (95–97), but the present results contribute to filling some gaps about these possible influences depending on participants' vaccination status, in a particular threatening context.

Considering that Romania was in the top 10 countries in Europe with the lowest vaccination rates against COVID-19, it is all the more important to investigate the underlying factors of Romanians' decision to get vaccinated (or not) to be able to act more effectively in future similar health-threatening situations. In addition, our results also highlighted the need for accurate information regarding the deadly nature of the virus, its severity, and the effectiveness of the COVID-19 vaccines to limit the influence of the COVID-19 infodemic and related deaths.

## Limitations and future directions

The present work has several limitations that need to be addressed. First, we used a convenience sample, and the number of participants was relatively low, which lowered the generalizability of the present findings (98). Furthermore, our sample was formed by young adults, which should also be considered when interpreting the present findings. For example, several previous studies highlighted the significance of age differences when discussing optimism bias regarding health-related behaviors [e.g., (25, 99)], as well as COVID-19 risk perception and preventive behaviors [e.g., (100)]. At the same time, the sample was unbalanced concerning the vaccination status, limiting both the generalizability and a more in-depth exploration regarding the possible moderating effects that we observed (i.e., the potential moderating role of age on the link between COVID-19 conspiracy beliefs and participants' anticipated regret for vaccinating, anticipated regret for non-vaccination, or subsequent regret—following vaccination). These concerns should be addressed in further studies using extended and more balanced samples of participants.

Also, it is important to mention that multivariate analysis was not conducted due to the skewed distribution of the vaccination intention/status variable, where some categories did not have large enough cell size distributions across exposures (COVID-19 conspiracy beliefs, COVID-19 threat perception, COVID-19 optimism bias, COVID-19 vaccination anticipated regret, and time spent online) and covariates (gender, age, and education level). Future studies might address this limitation by performing, for example, multivariate Ordinary Least Squares (OLS) or logistic regression using large enough cell size distributions.

Another limitation was the method of collecting the answers (i.e., online) and the self-reported character of the scales we used, which might have encouraged desirability. Also, optimism bias was only measured by the perceived personal risk of contracting SARS-CoV-2 but not the perceived threat to other people, and future studies might account for this in future approaches. Finally, another limitation could be represented by how we measured conspiratorial beliefs since we did not measure conspiratorial beliefs related to the vaccine's effectiveness or the dangers associated with it, but only conspiratorial beliefs related to the virus itself.

Future research could also explore the differences between those who have already been vaccinated and those who have not been vaccinated in terms of the perceived COVID-19 vaccine efficacy and risk, as these variables were important predictors in several studies and were not analyzed in the present work (14, 54, 79). In addition, future research might explore whether the restrictions adopted by the authorities and the introduction of the green certificate influenced the vaccination behavior of Romanians to some extent.

To conclude, the present study highlighted the significant association between optimism bias and some central variables within HBM and TBP theoretical models, such as the perceived threat and anticipated regret in the context of Anti-COVID-19 vaccination. Moreover, several significant differences depending on participants' vaccination intentions and status concerning the conspiracy beliefs highlighted the need for further studies in this area, especially given the uncertainty about the evolution of the COVID-19 pandemic.

## Data availability statement

The raw data supporting the conclusions of this article will be made available by the authors, without undue reservation.

## Ethics statement

The studies involving human participants were reviewed and approved by the Faculty of Psychology and Educational Sciences, Alexandru Ioan Cuza University. The patients/participants provided their written informed consent to participate in this study.

## Author contributions

All authors listed have made a substantial, direct, and intellectual contribution to the work and approved it for publication.

## Conflict of interest

The authors declare that the research was conducted in the absence of any commercial or financial relationships that could be construed as a potential conflict of interest.

## Publisher's note

All claims expressed in this article are solely those of the authors and do not necessarily represent those of their affiliated organizations, or those of the publisher, the editors and the reviewers. Any product that may be evaluated in this article, or claim that may be made by its manufacturer, is not guaranteed or endorsed by the publisher.
